# Ethical considerations and concerns in the implementation of AI in pharmacy practice: a cross-sectional study

**DOI:** 10.1186/s12910-024-01062-8

**Published:** 2024-05-16

**Authors:** Hisham E. Hasan, Deema Jaber, Omar F. Khabour, Karem H. Alzoubi

**Affiliations:** 1 Department of Clinical Pharmacy, Faculty of Pharmacy, Jordan University of Science and Technology, Irbid, 22110 Jordan; 2 Department of Clinical Pharmacy, Faculty of Pharmacy, Zarqa University, Zarqa, 13110 Jordan; 3 Department of Medical Laboratory Sciences, Faculty of Applied Medical Sciences, Jordan University of Science and Technology, Irbid, 22110 Jordan; 4 Department of Pharmacy Practice and Pharmacotherapeutics, College of Pharmacy, University of Sharjah, Sharjah, 27272 United Arab Emirates; 5 Faculty of Pharmacy, Jordan University of Science and Technology, Irbid, 22110 Jordan

**Keywords:** Artificial intelligence, Pharmacy practice, Ethical considerations, Technology literacy, MENA

## Abstract

**Background:**

Integrating artificial intelligence (AI) into healthcare has raised significant ethical concerns. In pharmacy practice, AI offers promising advances but also poses ethical challenges.

**Methods:**

A cross-sectional study was conducted in countries from the Middle East and North Africa (MENA) region on 501 pharmacy professionals. A 12-item online questionnaire assessed ethical concerns related to the adoption of AI in pharmacy practice. Demographic factors associated with ethical concerns were analyzed via SPSS v.27 software using appropriate statistical tests.

**Results:**

Participants expressed concerns about patient data privacy (58.9%), cybersecurity threats (58.9%), potential job displacement (62.9%), and lack of legal regulation (67.0%). Tech-savviness and basic AI understanding were correlated with higher concern scores (*p* < 0.001). Ethical implications include the need for informed consent, beneficence, justice, and transparency in the use of AI.

**Conclusion:**

The findings emphasize the importance of ethical guidelines, education, and patient autonomy in adopting AI. Collaboration, data privacy, and equitable access are crucial to the responsible use of AI in pharmacy practice.

**Supplementary Information:**

The online version contains supplementary material available at 10.1186/s12910-024-01062-8.

## Highlights

Pharmacy professionals in the MENA region express significant ethical concerns about integrating AI into pharmacy practice.

Key ethical considerations for AI highlighted in the current study include the privacy of patient data, AI replacing non-specialized pharmacists, and a lack of legal regulation.

Tech-savviness and basic understanding of AI are positively correlated with higher ethical concerns.

Informed consent as a vital part of autonomy, beneficence, and justice are crucial ethical principles in the adoption of AI in pharmacy.

Collaboration, education, and ethical frameworks are essential for the responsible use of AI in pharmacy practice.

**Supplementary Information:**

The online version contains supplementary material available at 10.1186/s12910-024-01062-8.

## Introduction

The integration of artificial intelligence (AI) into healthcare systems has launched a new era of transformative advancements in patient care and medical research [1]. Since the outbreak of the COVID-19 pandemic, the rapid adoption of AI technologies has been remarkable [2]. AI technologies have the potential to revolutionize how healthcare is delivered, offering innovative solutions from administrative workflows to improved patient outcomes [3]. However, this technological shift has raised significant concerns, particularly regarding the protection of individual privacy and the ethical implications associated with AI adoption in healthcare [4].

In pharmacy practice, the integration of AI holds great promise for many applications and innovations, including clinical decision support systems, automating medication dispensing, inventory management, detection of adverse drug reactions, and providing personalized medication regimens [5]. All these advancements can improve medication safety, enhance patient adherence, and increase overall pharmacy efficiency [5, 6]. Nevertheless, integrating AI into pharmacy practice presents a complex ethical landscape that requires careful consideration [6]. For example, concerns encompass the potential loss of empathy and trust in healthcare interactions, the risk of perpetuating biases in AI recommendations, and the complex technical and resource demands of AI systems [7, 8]. Nevertheless, wisely integrating AI as a complementary tool allows pharmacists to harness its benefits while upholding ethical standards and preserving the human element in healthcare [9].

Electronic health records often contain sensitive patient information, and protecting this data in AI programs is crucial, emphasizing the need for accurately labeled data to ensure reliable AI outcomes [10, 11]. Ethical concerns surround commercial access to AI outcomes, underscoring the importance of safeguarding patient privacy and preventing unethical data utilization [12]. Furthermore, AI algorithms should exhibit validity, reliability, and transparency, and the industry vendor must transparently define ownership, access, sharing, and patient data monitoring to guarantee privacy protection. Obtaining well-documented, informed patient consent is vital, particularly in research studies, rather than relegating consent language to ambiguous terms and conditions [13, 14].

Ethical concerns in adopting AI are paramount, as it ensures individual rights, protection of patient data, and adherence to principles of biomedical ethics [15]. This study aims to delve into the ethical dimensions surrounding the integration of AI into pharmacy practice, with a particular focus on the MENA region. It seeks to explore the perspectives of various pharmacy professionals, including licensed pharmacists and pharmacy faculty members, regarding ethical considerations associated with implementing AI. It also assesses variations of these concerns in the pharmacy ecosystem, examines their implications, and contributes to the discourse on healthcare ethics in the age of AI.

Through empirical analysis, this study delves into the sentiments of pharmacy practice participants regarding the ethical implications of AI integration. The intersection of AI and biomedical ethics is a relatively unexplored area, and this research bridges the gap between technological advancement and ethical considerations.

## Materials and methods

### Study design

This cross-sectional study employed a validated online questionnaire hosted on the Google Forms^®^ platform. Questionnaire items were developed for this study by H.E.H and D. J., with the assistance of experts in pharmacy practice research and based on a literature review [5, 13, 16]. The questionnaire was divided into two parts (Additional file 1). The first part was designed to obtain the sociodemographic characteristics of the respondents, including gender, age, income, educational level, etc. The second part included 12 items and investigated ethical considerations and concerns related to integrating AI into daily pharmacy practice in the MENA. The Likert’s agreement five-point response scale (strongly disagree, which scores 1 point; strongly agree, which scores 5 points) was used. The survey, validated from a previously published research protocol [17], was conducted over six months, from August 2022 to January 2023.

### Sample size and selection strategy

The minimum sample size required was calculated via Raosoft^®^, using a 95% confidence level and a 5% margin of error (significance α = 0.05) with a 50% response distribution. It was found to be 384 subjects. The target population for this survey was pharmacists across seven countries, including Jordan, Libya, Egypt, Lebanon, Saudi Arabia, Kuwait, and Palestine. Participants were selected through convenience sampling based on their connection to the field of pharmacy. Inclusion criteria include licensed pharmacists and pharmacy faculty members residing in the MENA regions.

### Recruitment and data collection

The potential subjects were informed that participation is voluntary and they could withdraw without penalty. No incentives were provided to the participants to maintain the voluntary nature of their participation. The questionnaire was distributed electronically to a diverse group of participants who have a stake in the field of pharmacy practice, including pharmacists and faculty members, through online channels, including e-mail and social media platforms (such as WhatsApp^®^ and Facebook^®^ groups). The survey was administered in both English and Arabic to accommodate the linguistic diversity of the participants. The anonymity of participants was maintained to encourage open and honest responses.

### Ethical considerations

The study was conducted in accordance with ethical guidelines and principles. Ethical approval was obtained from the Research Ethics Committee at Zarqa University (Approval No. 54/2021/2022). An electronic informed consent was obtained from all participants, which outlined the purpose of the study and emphasized the voluntary nature and anonymity of their participation.

### Data analysis

Statistical analyses were performed using the Statistical Package for Social Science (IBM^®^ SPSS^®^) software, version 27. Data analysis was conducted in two main stages: demographic data and ethical concern questions were summarized using descriptive statistics. The mean, standard deviation, frequencies, and percentages were calculated to provide an overview of the sample characteristics. Various inferential statistical tests were employed to identify significant variations within the study groups, including the independent *t-*test, one-way analysis of variance (ANOVA), Pearson’s correlation (*r*), multiple linear regression, and logistic regression. Odds ratios (OR) and 95% confidence intervals (CI) were calculated for each predictor variable. The questionnaire demonstrated high internal consistency and reliability of the Likert-scale items, with a Cronbach’s alpha (*α*) score of 0.916. A significance level of *p* < 0.05 was set to determine statistical significance in all tests.

## Results

A total of 501 participants volunteered for this study, representing a diverse set of demographic characteristics, as shown in Table 1. The largest group of participants resided in Lebanon (31.1%), followed by Jordan (18.2%) and Libya (16.2%). Most respondents were working as pharmacists (65.5%) or faculty members (34.5%) in colleges of pharmacy. The average age of the participants was 36.7 ± 10.8 years. The majority of participants were females (61.9%) with a diverse range of marital statuses. Household net income is distributed across the lower class (20.6%), middle class (37.7%), and upper class (41.7%). Participants belonged to or were affiliated with the government (59.9%) and private (40.1%) sectors. Educational level or academic degree varied, with a significant number holding a bachelor’s degree in pharmacy (51.1%). The work status ranged from not working (11.2%) to full-time employment (59.1%). The workplaces included community pharmacies (28.7%), university settings (34.5%), and others.

**Table 1:** Demographic characteristics of study participants (*N* = 501)

Variables	Categories	*N* (%)
Country of residence	Jordan	91 (18.2)
	Libya	81 (16.2)
	Lebanon	156 (31.1)
	Egypt	88 (17.6)
	Palestine	23 (4.6)
	Saudi Arabia	32 (6.4)
	Kuwait	30 (6.0)
Occupation	Pharmacist	328 (65.5)
	Faculty member	173 (34.5)
Gender	Male	191 (38.1)
	Female	310 (61.9)
Marital status	Single	172 (34.3)
	Married	299 (59.7)
	Others	30 (6.0)
Monthly income^a^	Lower class	103 (20.6)
	Middle class	189 (37.7)
	Upper class	209 (41.7)
Sector	Governmental	300 (59.9)
	Private	201 (40.1)
Educational level	BPharm	256 (51.1)
	PharmD	66 (13.2)
	MSc	73 (14.6)
	PhD	106 (21.2)
Work status	Not working^b^	56 (11.2)
	Full-time	296 (59.1)
	Part-time	92 (18.4)
	Self-employed	57 (11.4)
Workplace^*^	Community pharmacy	144 (28.7)
	Hospital pharmacy	26 (5.2)
	Drug store or company	16 (3.2)
	University	173 (34.5)
	Others	15 (3.0)
Tech-savviness	Strongly disagree	39 (7.8)
	Disagree	76 (15.2)
	Neutral	171 (34.1)
	Agree	136 (27.1)
	Strongly agree	79 (15.8)
Basic AI understanding	Strongly disagree	112 (22.4)
	Disagree	129 (25.7)
	Neutral	149 (29.7)
	Agree	68 (13.6)
	Strongly agree	43 (8.6)

^a^ Income categories are based on World Bank classification data for 2022

^b^ The unemployed refers to licensed pharmacists who are not currently working in a specific position but may be in transition due to various reasons such as vacation, job searching, recent graduation, or retirement

^*^ This question was optional and allowed responses from those with active working status

Table 2 presents responses regarding participants’ concerns and issues regarding the integration of AI into pharmacy practice. Participants expressed concerns about the risks to patient data privacy, with 58.9% agreeing. The majority of participants (58.9%) were concerned about pharmacy AI systems being vulnerable to hacking and cybersecurity threats. About 62.9% agreed that AI systems may replace non-specialized pharmacists. Concerns about costly subscriptions limiting accessibility were shared by 63.7% of participants. Participants also raised concerns about the lack of access to AI technologies, with 67.6% agreeing that it poses a barrier to pharmacy practice. About two-thirds (67.0%) of participants expressed concern about the absence of comprehensive legal regulation for AI in pharmacy practice. Similarly, 68.8% of participants believed there was a lack of proper training for pharmacists to effectively use AI in practice. Concerns about physicians’ reluctance to embrace AI were shared by 62.4% of participants. 60.3% agreed that patients are apprehensive about AI’s ability to create suitable treatment plans. Participants (63.5%) agreed that AI may affect the time allocated for patient counseling due to its limited communication skills and lack of body language. 56.5% were concerned about AI systems overselling unnecessary over-the-counter medications and cosmetics to patients. A significant majority (59.4%) agreed that educating AI developers about data privacy and ethics is essential for the responsible integration of AI in healthcare.

**Table 2:** Concerns and issues related to AI in pharmacy practice (*N* = 501)

No.	Statements		*N* (%)		M ± SD
		Disagree/Strongly Disagree	Neutral	Agree/Strongly Agree	
**1**	To what extent do you agree with the statement that AI in pharmacy practice poses a risk to patient data privacy?	55 (11.0)	151 (30.1)	295 (58.9)	3.68 ± 1.001
**2**	How concerned are you about AI systems in pharmacies being vulnerable to hacking and cybersecurity threats?	47 (9.4)	159 (31.7)	295 (58.9)	3.72 ± 0.985
**3**	To what extent do you believe that AI systems may replace non-specialized pharmacists in pharmacy practice?	47 (9.4)	139 (27.7)	315 (62.9)	3.75 ± 0.968
**4**	Do you think that the requirement of costly subscriptions for AI systems limits their accessibility?	30 (6.0)	152 (30.3)	319 (63.7)	3.77 ± 0.879
**5**	To what extent do you agree that the lack of access to AI technologies is a barrier in pharmacy practice?	29 (5.8)	133 (26.5)	339 (67.7)	3.88 ± 0.934
**6**	How concerned are you about the absence of comprehensive legal regulation for AI in pharmacy practice?	27 (5.4)	138 (27.5)	336 (67.1)	3.88 ± 0.906
**7**	Do you believe that there is a lack of proper training for pharmacists to effectively use AI in practice?	27 (5.4)	129 (25.7)	345 (68.9)	3.92 ± 0.906
**8**	To what extent do you agree that physicians are reluctant to embrace AI in pharmacy practice?	34 (6.8)	154 (30.7)	313 (62.5)	3.80 ± 0.924
**9**	Do you believe patients are apprehensive about AI’s ability to create suitable treatment plans?	38 (7.6)	161 (32.1)	302 (60.3)	3.72 ± 0.928
**10**	To what extent do you agree that AI may affect the time allocated for patient counseling due to its limited communication skills and lack of body language?	38 (7.6)	145 (28.9)	318 (63.5)	3.76 ± 0.905
**11**	How concerned are you about the potential for AI systems to oversell unnecessary over-the-counter medications and cosmetics to patients?	51 (10.2)	167 (33.3)	283 (56.5)	3.63 ± 0.972
**12**	To what extent do you agree that educating AI developers about data privacy and ethics is essential for the responsible integration of AI in healthcare?	89 (17.8)	116 (26.1)	296 (59.1)	3.73 ± 1.272

Table 3 presents a comprehensive analysis of various parameters and their influence on the total concern score. It offers valuable insights into the interplay of different demographic and contextual variables in shaping individuals’ AI-related concerns. The country of residence emerged as a significant factor influencing the total concerns. Marital status showed statistical significance, with married individuals expressing higher concerns. Workplace significantly impacted concerns, with individuals working in drug stores or companies showing the highest concern scores. Participants who strongly agreed with their tech-savviness and basic AI understanding displayed the highest mean total concern scores, with both variables being highly significant. Other factors, such as occupation, gender, sector, income, academic degree, and work status, did not significantly impact concerns.

**Table 3:** Parameters affecting the mean total AI-concerns score

Variable	Categories	Mean Total Concerns Score (%) ± SD	*p*-value^*^
Country of residence	Jordan	74.8 ± 12.1	**0.005**
	Egypt	75.4 ± 12.4	
	Lebanon	75.3 ± 14.0	
	Libya	80.1 ± 11.7	
	Palestine	76.8 ± 8.8	
	Saudi Arabia	70.5 ± 9.6	
	Kuwait	75.5 ± 10.1	
Occupation	Pharmacist	74.9 ± 14.8	0.239
	Faculty members	76.3 ± 12.1	
Gender	Males	74.4 ± 13.8	0.225
	Females	76.0 ± 14.0	
Material status	Single	75.9 ± 12.1	**0.003**
	Married	76.2 ± 12.6	
	Others	72.7 ± 13.1	
Monthly income	Lower class	76.0 ± 12.0	0.787
	Middle class	75.2 ± 12.4	
	Upper class	76.5 ± 12.7	
Sector	Governmental	75.4 ± 14.0	0.898
	Private	75.3 ± 13.8	
Educational level	Bachelor’s	76.4 ± 12.1	0.648
	PharmD	72.7 ± 14.9	
	Master’s	75.6 ± 12.8	
	PhD	76.8 ± 11.6	
Work status	Not working	92.2 ± 4.2	0.051
	Full-time	74.9 ± 12.5	
	Part-time	77.1 ± 10.4	
	Self-employed	78.6 ± 14.2	
Workplace	Community pharmacy	74.3 ± 12.7	**0.007**
	Hospital pharmacy	74.7 ± 12.6	
	Drug store or company	86.0 ± 8.6	
	University	76.4 ± 12.1	
	Others	78.1 ± 13.5	
Tech-savviness	Strongly disagree	68.0 ± 16.2	**< 0.001**
	Disagree	73.2 ± 12.9	
	Neutral	74.6 ± 11.1	
	Agree	77.3 ± 12.3	
	Strongly agree	81.3 ± 11.4	
Basic AI understanding	Strongly disagree	70.8 ± 14.9	**< 0.001**
	Disagree	75.2 ± 12.0	
	Neutral	77.0 ± 10.8	
	Agree	78.4 ± 10.5	
	Strongly agree	82.0 ± 12.5	

^*^ A *p-*value of less than 0.05 indicates statistical significance, calculated by either an independent *t-*test or ANOVA when appropriate

Table 4 presents the correlation analysis of the total concern score. It demonstrates that the total concern score exhibits a significant positive correlation with tech-savviness (*r* = 0.345, *p* < 0.001) and basic AI understanding (*r* = 0.284, *p* < 0.001). These findings suggest that individuals with higher tech-savviness and AI understanding tend to report higher concerns. Conversely, there is a negative correlation between the total concern score and both age (*r* = -0.082) and experience (*r* = -0.105). Although this relationship is not statistically significant (*p* = 0.068) for age, it is statistically significant (*p* = 0.018) for experience, implying that concerns tend to decrease slightly as experience increases. Tables 5 and 6 highlight multiple linear regression and logistic regression analyses, respectively, that investigated the impact of these various factors on the total concerns related to AI integration in the pharmaceutical field. The regression model, as indicated by the F-value (*F* = 19.371, *p* < 0.001), is statistically significant, demonstrating its ability to explain a portion of the variance in concerns. The R-squared value (*R*^*2*^ = 0.135) suggests that age, experience, tech-savviness, and AI understanding collectively account for 13.5% of the variance in the total concern scores. Further analysis demonstrates that the regression model significantly explains the variance in the total concern score (*p* < 0.001). Regression coefficients highlight the individual contributions of age, experience, tech-savviness, and AI understanding. Tech-savviness exerts the most substantial influence with a standardized coefficient (*β*) of 0.266, followed by AI understanding (*β* = 0.123). Age has a smaller but significant effect (*β* = 0.069), while experience (*β* = -0.133) indicates lower-year experienced individuals tend to have slightly higher total concern scores. Gender was found to be a significant predictor of heightened concern among participants. Males had a lower odds ratio (OR = 0.574, 95% CI: 0.339–0.975, *p* = 0.040) than females (reference category). Individuals working in governmental sectors were more likely to have high concerns than those in private sectors (OR = 1.883, 95% CI: 1.062–3.336, *p* = 0.030). Participants with a PharmD or master’s degree were more likely to have low concerns compared to those with a PhD (reference category). The odds ratios were 0.218 (95% CI: 0.061–0.781, *p* = 0.019) for PharmD and 0.394 (95% CI: 0.175–0.890, *p* = 0.025) for MSc. Individuals who strongly disagreed with being tech-savvy and having a basic understanding of AI were less likely to have significant concerns (OR = 0.197, 95% CI: 0.041–0.950, *p* = 0.043), (OR = 0.181, 95% CI: 0.051–0.649, *p* = 0.009).

**Table 4:** Correlation analysis of independent variables with the total concerns score

Variable	M ± SD	Pearson Correlation Coefficient (*r*)	*p*-value^*^
Age (Years)	36.7 ± 10.8	− 0.082	0.068
Experience (Years)	12.9 ± 10.0	− 0.105	**0.018**
Tech-savviness	3.3 ± 1.1	0.345	**< 0.001**
Basic AI understanding	2.6 ± 1.2	0.284	**< 0.001**
Total concerns score out of 100%	75.4 ± 13.9	1	-

^*^ A *p-*value of less than 0.05 indicates statistical significance, calculated by Pearson’s r

**Table 5:** Summary of the overall regression model analysis results

Dependent Variable	*R*^2^	F-Statistic	*p*-value^*^	Significant Predictors
Total concern score	0.135	19.371	**< 0.001**	Age, experience, Tech-savviness, basic AI understanding

^*^ A *p-*value of less than 0.05 indicates statistical significance, calculated by ANOVA’s linear regression

**Table 6:** Logistic regression analysis results

Predictor	Sub-Categories	OR	95% CI. for OR		*p*-value^*^
			Lower	Upper	
Country of residence	Jordan	0.842	0.279	2.541	0.760
	Libya	0.946	0.288	3.106	0.928
	Lebanon	1.055	0.331	3.366	0.928
	Egypt	0.733	0.239	2.249	0.587
	Palestine	0.988	0.232	4.202	0.987
	Kuwait (REF)	-	-	-	0
Occupation	Pharmacist	0.000	0.000	-	1.000
	Faculty member (REF)	-	-	-	0
Gender	Male	0.574	0.339	0.975	**0.040**
	Female (REF)	-	-	-	0
Age		1.037	0.958	1.124	0.368
Marital status	Single	1.717	0.487	6.054	0.400
	Married	1.329	0.406	4.343	0.638
	Others (REF)	-	-	-	0
Monthly income	Lower class	1.957	0.841	4.554	0.119
	Middle class	1.224	0.651	2.304	0.530
	Upper class (REF)	-	-	-	0
Sector	Governmental	1.883	1.062	3.336	**0.030**
	Private (REF)	-	-	-	0
Educational level	BPharm	0.357	0.120	1.068	0.066
	PharmD	0.218	0.061	0.781	**0.019**
	MSc	0.394	0.175	0.890	**0.025**
	PhD (REF)	1.000	-	-	0
Work status	Not working	∞	-	-	1.000
	Full-time	0.316	0.128	0.782	**0.013**
	Part-time	0.461	(0.167	1.271	0.134
	Self-employed (REF)	-	-	-	0
Workplace	Community pharmacy	0.866	0.231	3.243	0.831
	Hospital pharmacy	1.109	0.229	5.372	0.897
	Drug store or company	∞	-	-	1.000
	University	∞	-	-	1.000
	Others (REF)	0.000	-	-	1.000
Experience		0.977	0.903	1.057	0.555
Tech-savviness	Strongly disagree	0.197	0.041	0.950	**0.043**
	Disagree	0.397	0.139	1.140	0.086
	Neutral	0.527	0.196	1.146	0.097
	Agree	0.617	0.262	1.453	0.269
	Strongly agree (REF)	0.594	0.176	2.001	0.401
Basic AI understanding	Strongly disagree	0.181	0.051	0.649	**0.009**
	Disagree	0.358	0.108	1.192	0.094
	Neutral	0.501	0.160	1.565	0.234
	Agree	0.594	0.176	2.001	0.401
	Strongly agree (REF)	0.594	0.176	2.001	0.401

^*^ A *p-*value of less than 0.05 indicates statistical significance

## Discussion

Integrating AI into pharmacy practice is a promising avenue for innovation and enhanced patient care, but it also raises essential ethical considerations [6, 7]. This study aimed to explore the ethical dimensions of AI adoption in pharmacy practice in the MENA region and investigate the concerns and perspectives of pharmacy professionals. The results provide valuable insights into the potential challenges associated with the use of AI, framed within the principles of biomedical ethics, which include autonomy, beneficence, non-maleficence, and justice.

### Concerns related to AI integration

#### Autonomy

One of the primary ethical concerns surrounding AI in pharmacy practice is the protection of patient autonomy and informed consent. Many participants expressed concerns about patients’ apprehension regarding AI’s ability to create suitable treatment plans. While this study examines the perspectives of pharmacy practice experts, it is essential to recognize that patient perspectives were similar in existing literature, which investigated patients’ apprehensions regarding AI in healthcare, emphasizing concerns related to safety, autonomy, and higher costs as factors influencing patient acceptance [18]. This raises questions about patients’ ability to make informed choices about their healthcare when it comes to AI systems. Patients should have the autonomy to decide whether or not they are comfortable with AI-driven recommendations and understand the implications. It is an obligation to provide patients with clear information about AI use, ensuring trust and informed decisions regarding their treatment plans. Moreover, nearly two-thirds of participants (62.4%) expressed concerns about physician reluctance to embrace AI, aligning with the broader sentiment observed in a systematic review where over 60% of physicians and medical students displayed positive yet reserved attitudes toward clinical AI, indicating a cautious approach despite increasing awareness of AI and its clinical applications [19]. However, more recent qualitative study findings highlighted low awareness and engagement among doctors working with AI in United Kingdom healthcare [20]. These concerns emphasize the importance of fostering trust and acceptance of AI technologies among healthcare professionals and patients. Ethical communication and transparency in AI implementation can help address these concerns and build confidence in AI-assisted healthcare. In addition to ethical considerations surrounding patient autonomy, it’s essential to address confidentiality issues, which directly intersect with autonomy. However, due to the serious harm of potential confidentiality breaches, further discussion is in the following section.

#### Beneficence and non-maleficence

The ethical principles of beneficence and non-maleficence require healthcare professionals to act in the best interests of their patients and avoid harm. In our context, over half of the participants (56.5%) had concerns about AI systems potentially overselling unnecessary medications and cosmetics to patients, raising questions about beneficence and potential maleficence. Such an act may be the result of a biased algorithmic system in the pharmaceutical market, and the absence of legally binding regulations in such a situation would harm consumers [21]. AI in pharmacy practice should prioritize patient well-being and ensure that recommendations are based on evidence-based guidelines rather than profit motives. Pharmacy professionals must critically evaluate AI-driven recommendations to ensure they align with patients’ best interests.

Additionally, the study revealed concerns about the impact of AI on patient counseling due to its limited communication skills and lack of body language. This is a significant ethical consideration, as it touches on the potential harm to the patient-provider relationship. Despite AI’s potential benefits in disease detection during the COVID-19 pandemic, our findings align with concerns raised in the literature in this regard and the need to ensure a positive impact on person-centered care [22, 23]. Especially in the deployment of mental health chatbots [24]. Pharmacy professionals must ensure that AI does not compromise the quality of care provided to patients. They must balance the efficiency gains AI offers with maintaining the human touch in patient interactions.

One primary concern in pharmacy practice was the risk to patient data privacy. Almost 60% of participants were apprehensive that AI threatened data privacy. Health data, unlike other types of data, is highly personal and confidential and might affect individuals’ health, well-being, and personal lives. Its sensitivity extends to the risk of shame, stigma, or discrimination, a concern particularly prevalent in developing countries [25, 26]. As AI systems typically involve collecting, storing, and analyzing sensitive patient information, these concerns are valid [4]. This underscores the need for robust data protection measures and adherence to privacy regulations such as the Health Insurance Portability and Accountability Act (HIPAA) in the United States [27, 28]. In addition, the study highlighted worries about AI systems in pharmacies being vulnerable to hacking and cybersecurity threats. Over 58% of participants agreed with this concern. With the rise in cyberattacks on healthcare systems, as emphasized in a recent study, securing AI infrastructure is of paramount importance [29]. Potential patient confidentiality breaches are a primary ethical concern, and pharmacy stakeholders should prioritize data protection and security when implementing AI technologies. Investing in such cybersecurity solutions is essential to safeguarding data and maintaining trust between healthcare providers and patients [30].

Furthermore, a significant portion of the participants (62.9%) believed that AI systems may replace non-specialized pharmacists. This raises ethical questions about its impact on employment within the pharmacy sector, which led to workforce displacement. The fear of job displacement due to automation is not unique to the pharmacy field alone but resonates across various sectors. A different pattern was noticed in a previous study, where the majority disagreed that pharmacists could be replaced [31]. This might be because we asked about ‘non-specialized pharmacists’ (not certified by the Board of Pharmacy Specialties^®^) and not the pharmacy profession. It is crucial to recognize that while AI can automate specific tasks, it can also enhance the capabilities of pharmacists, enabling them to focus on more complex, patient-centered aspects of their roles. Therefore, the integration of AI should be seen as a tool to augment and complement the skills of pharmacy professionals, rather than replace them. Similarly, in alignment with Arab Muslim societies, the belief that God is the sole sustainer coexists with the understanding that individuals must be proactive and exert morally reasonable effort to acquire their provision [32]. Ethical considerations must encompass strategies for retraining and reskilling pharmacy professionals most affected by these changes, ensuring they remain relevant and valuable in the evolving landscape of pharmacy practice.

#### Justice

The principle of justice emphasizes fair and equitable access to healthcare resources and benefits. The study highlighted concerns about the cost of AI subscriptions (63.7%) and the lack of accessibility (67.6%), posing barriers to pharmacy practice. This highlights the potential for AI to exacerbate healthcare disparities. It is essential to ensure that AI technologies are accessible to all, regardless of economic status or location [7, 31]. Ethical frameworks must be developed to address these concerns and promote equitable access to AI-driven healthcare tools. To ensure this, governments, healthcare organizations, and technology providers must work together to develop pricing models that do not exclude smaller or underfunded healthcare settings. The financial resources of the institution should not determine accessibility to AI technologies but rather be based on their clinical and patient care needs.

#### Legal regulation, education, and training

The concerns raised by participants regarding the lack of comprehensive legal regulation for AI use in current pharmacy practice (67.0%) and the need to educate AI developers about data privacy and ethics (59.4%) are intertwined. Ethical considerations encompass data privacy and the responsible development and deployment of AI technologies, which emphasizes the importance of educating AI developers about biomedical ethics principles. They must understand the ethical implications of their technologies and prioritize patient well-being. It is also essential for governments and professional bodies to establish clear regulations and ethical frameworks that guide the safe and effective use of AI in healthcare. A recently published meta-analysis of 200 policies and guidelines for AI usage aligns with our study, with no representation of the MENA region, and emphasizes the global need for a unified approach in shaping future regulations to guide the ethical use of AI across diverse domains, including healthcare and pharmacy practice [33]. Moreover, collaboration between healthcare professionals, ethicists, and AI developers is crucial to ensuring that AI systems are designed, deployed, and adhere to these ethical guidelines.

The ethical considerations highlighted in prior research on AI in medical education align with our findings, emphasizing the need for regulations and education to ensure responsible AI development and transparent implementation [34]. Almost 70% of participants believed there was a lack of proper training for pharmacists to effectively use AI in practice. Pharmacy professionals are ethically obligated to maintain their competence and continue their education. Adequate training programs should be established to prepare pharmacists with the skills and knowledge needed to use AI technologies effectively and ethically. Educational institutions should collaborate with other sectors to develop curricula and training programs that equip future pharmacists with the necessary workflow skills.

### Factors influencing concerns

Participants from different countries in the MENA region had varying levels of concern (*p* = 0.005). This is in line with the observed regional disparities in the findings of a review article that identified challenges and opportunities in MENA’s health systems, such as financial, organizational, and behavioral factors [35]. Also, the digital health technologies in less fortunate and conflict-affected areas of the MENA region have been explored and revealed some considerations for the adoption of digital health, such as computer literacy, weak technological infrastructure, and privacy concerns, which align with the acknowledgment of varying concerns among participants from different MENA regions [36]. Both discussions suggest the importance of considering regional, cultural, and contextual factors in shaping attitudes toward AI in pharmacy practice to address possible challenges in diverse settings. Less experienced individuals tended to have slightly higher concerns (*r* = -0.105, *p* = 0.018). Married individuals expressed higher concerns (*p* = 0.003), which could be related to the specific considerations and responsibilities associated with their status. These findings highlight the need for tailored strategies to address the concerns of different demographic groups, as these groups may perceive themselves as more vulnerable to being replaced with AI. Participants working in specific settings, such as drug stores or companies, reported higher concerns (*p* = 0.007). A drug store or company operates in a different professional setting from a community pharmacy, with a focus beyond direct patient care and medication dispensing. It encompasses various roles and functions related to the managerial, operational, and strategic aspects of the pharmaceutical industry [37]. These include marketing, business development, quality assurance, formulation, regulatory affairs, and other related tasks. This suggests that the institutional and workplace contexts can significantly impact attitudes toward AI integration, and participants affiliated with these places might have more interaction with new technologies such as AI, virtual reality, and robotics, leading to distrust and, eventually, fewer work opportunities [38]. Participants with higher levels of tech-savviness and basic AI understanding reported higher concerns (*p* < 0.001 for both). This finding underscores the importance of technology literacy and education in shaping attitudes and concerns related to AI. A similar trend was observed in a Jordanian study about using an AI-powered chatbot, namely ChatGPT, in pharmacy, with higher concerns strongly associated with more awareness [39]. Further analysis revealed that tech-savviness and basic AI understanding were positively correlated with higher concern scores. Age exhibited a negative but weak correlation and a relatively small effect on overall concerns. These findings suggest that individuals with better technology and AI literacy tend to have greater concerns about AI integration, as they carefully examined AI systems and might be more relevant and oriented with the complexity behind its adoption, advanced issues surrounding it, and potential misuse of such technology. Pharmacy professionals who are more familiar with AI technology may have a competitive advantage in future pharmacy practice settings [40]. These identified predictors provide valuable insights for educational institutions and policymakers and emphasize the importance of promoting AI literacy and awareness to facilitate informed ethical decision-making.

### Implications of the findings

The insights garnered from this study hold significant implications for informing policy, shaping practice, and guiding education in the field. It is crucial to develop strategies that foster trust and enhance transparency when implementing AI. One practical recommendation is implementing educational programs for healthcare professionals and patients. These AI literacy programs can demystify these technologies, clarify their role in treatment plans, and address misconceptions. Additionally, creating user-friendly interfaces that explain AI-driven recommendations in plain language can empower patients to make more informed decisions about their healthcare. To overcome pharmacy professionals’ and healthcare providers’ reluctance, targeted interventions are needed. This includes incorporating AI education into medical curricula, providing hands-on training opportunities, and developing guides on effectively communicating AI’s role in treatment plans to ensure informed consent and trust. Policymakers can use these insights to develop robust regulations based on global best practices. Such policies should safeguard patient confidentiality and establish consequences for breaches. Acknowledging and actively addressing these concerns through powerful regulatory frameworks, stringent data privacy measures, and ongoing algorithmic audits can help build a more ethical foundation for AI implementation in pharmacy practice.

### Limitations of the study and future research

The sample size may not represent the entire pharmacy community, limiting the generalizability of the findings. Additionally, the survey methodology relied on self-reported concerns, which could be subject to response bias. Future studies could benefit from more diverse and larger samples. Future research could conduct in-depth interviews to gain a more comprehensive understanding of the ethical concerns and perspectives of pharmacy professionals and stakeholders. Longitudinal studies could track changes in attitudes and concerns over time as AI becomes more integrated into pharmacy practice. Comparative studies across different countries could highlight variations in concerns and the readiness of varying healthcare systems for AI integration. They are investigating the effectiveness of specific interventions, such as education programs, in mitigating ethical concerns and improving the responsible implementation of AI in pharmacy practice.

In conclusion, this study offers important insights into the ethical considerations of integrating AI into pharmacy practice in the MENA region. Patient autonomy, beneficence, non-maleficence, and justice emerge as critical guiding principles, emphasizing the need for responsible adoption that prioritizes patient welfare, data security, and accessibility. The results underscore the need for robust ethical frameworks, regulatory guidelines, and educational initiatives. Collaboration among healthcare professionals, AI developers, and regulatory bodies is essential to developing ethical guidelines and policies prioritizing patient interests and preserving the human element in pharmacy practice. As AI continues to transform the future of healthcare, addressing these ethical concerns is essential for maintaining trust and integrity in the evolving landscape of AI integration, requiring ongoing research and collaborative endeavors to uphold the highest ethical standards.

## Conclusion

This study has revealed the multifaceted landscape of AI integration in pharmacy practice, underscoring significant concerns and potential benefits. The findings emphasize the pressing need for ethical guidelines and regulatory frameworks that protect patient data privacy, ensure cybersecurity, and promote equitable access to AI systems. As the pharmacy profession navigates the transformative power of AI, future research endeavors should focus on innovative solutions, educational strategies, and collaborative models that maximize the advantages of AI while safeguarding patient welfare and the ethical principles inherent to biomedical practice.

### Electronic supplementary material

Below is the link to the electronic supplementary material.

Supplementary Material 1

Supplementary Material 2

## Data Availability

The dataset supporting the conclusions of this article is included within the article and its additional file.

## Abbreviations

AI: Artificial intelligence
MENA: Middle East and North Africa
