# Supplementary material for: Ethical considerations and concerns in the implementation of AI in pharmacy practice: a cross-sectional study
Source: BMC Med Ethics. 2024 May 16;25:55. doi: 10.1186/s12910-024-01062-8 (PMC11096093; doi:10.1186/s12910-024-01062-8)
Supplement: Supplementary file 1 — Supplementary Material 1 [file 12910_2024_1062_MOESM1_ESM.docx]

**Questionnaire**

Welcome to our research survey on the ethical considerations and perspectives regarding the integration of artificial intelligence (AI) in pharmacy practice. This survey is a part of a research project aimed at better understanding the concerns and issues related to AI in the pharmacy ecosystem.

Before you proceed with the survey, we would like to inform you about the nature of this research and seek your informed consent. Please read the following information carefully:

- This survey is designed to collect data on your opinions, concerns, and perspectives related to AI in pharmacy practice and biomedical ethics. Your input will contribute to a better understanding of the ethical implications of AI integration in pharmacy and its potential impact on patient care.
- Your participation in this survey is entirely voluntary. You are under no obligation to complete the survey, and you may withdraw at any time without consequence.
- All responses will be kept confidential. Your individual responses will not be shared or associated with your personal identity. Data will be analyzed and reported in aggregate form to ensure anonymity.
- The survey will take approximately 5 minutes to complete.
- If you have any questions or concerns about this survey or your participation, please contact [hehassan23@ph.just.edu.jo](mailto:hehassan23@ph.just.edu.jo).

Thank you for your time and valuable input.

1. Are you willing to participate in this survey?

- Yes
- No

**Section 1: Sociodemographic**

2. Country of Residence:

- Jordan
- Libya
- Lebanon
- Egypt
- Palestine
- Saudi Arabia
- Kuwait

3. Occupation:

- Pharmacist
- Faculty Member

4. Gender:

- Male
- Female

5. Age (Years):

- Filling by English number: ________

6. Experience (Years):

- Filling by English number: ________

7. Marital Status:

- Single
- Married
- Others

8. Monthly Income:

- Lower Class
- Middle Class
- Upper Class

9. Sector:

- Governmental
- Private

10. Academic Degree:

- Bachelor of Pharmacy (BPharm)
- Doctor of Pharmacy (PharmD)
- Master of Sciences of Pharmacy (MPharm)
- Doctor of Philosophy (PhD)

11. Work Status:

- Not Working
- Full-Time Employment
- Part-Time Employment
- Self- Employment

12. Workplace:

- Community Pharmacy
- Hospital
- Drug Store / Company
- University
- Others

13. I consider myself a tech-savvy (well informed about or proficient in the use of modern technology, especially computers).

1 2 3 4 5

Strongly disagree Strongly agree

14. I have a basic understanding of AI technology in pharmacy.

1 2 3 4 5

Strongly disagree Strongly agree

**Section 2: Ethical Concerns**

1 2 3 4 5

Strongly disagree Strongly agree

- To what extent do you agree with the statement that AI in pharmacy practice poses a risk to patient data privacy?
- How concerned are you about AI systems in pharmacies being vulnerable to hacking and cybersecurity threats?
- To what extent do you believe that AI systems may replace non-specialized pharmacists in pharmacy practice?
- Do you think that the requirement of costly subscriptions for AI systems limits their accessibility?
- To what extent do you agree that the lack of access to AI technologies is a barrier in pharmacy practice?
- How concerned are you about the absence of comprehensive legal regulation for AI in pharmacy practice?
- Do you believe that there is a lack of proper training for pharmacists to effectively use AI in practice?
- To what extent do you agree that physicians are reluctant to embrace AI in pharmacy practice?
- Do you believe that patients are apprehensive about AI's ability to create suitable treatment plans?
- To what extent do you agree that AI may affect the time allocated for patient counseling due to its limited communication skills and lack of body language?
- How concerned are you about the potential for AI systems to oversell unnecessary over-the-counter medications and cosmetics to patients?
- To what extent do you agree that educating AI developers about data privacy and ethics is essential for the responsible integration of AI in healthcare?

Thank you for your contribution and patience.
